# Investigating the Role of Coenzyme A Restriction in the Pathophysiology of Preeclampsia: Protocol for a Combined Patient Screening and Laboratory Study

**DOI:** 10.2196/66202

**Published:** 2025-10-03

**Authors:** Reem Al-Jayyousi, Reem K Jan, Alexander D Giddey, Adrian G Stanley, Anagha Parambath, Fadi G Mirza, Rajan Radhakrishnan, William Atiomo, Charlie Hodgman

**Affiliations:** 1 College of Medicine Mohammed Bin Rashid University of Medicine and Health Sciences Dubai United Arab Emirates; 2 Center for Applied and Translational Genomics Mohammed Bin Rashid University of Medicine and Health Sciences Dubai United Arab Emirates; 3 Mediclinic Middle East Dubai United Arab Emirates; 4 Latifa Women and Children Hospital Dubai United Arab Emirates; 5 School of Biosciences University of Nottingham Nottingham United Kingdom

**Keywords:** preeclampsia, coenzyme A restriction, patient samples, cell culture study, high-performance liquid chromatography-mass spectrometry

## Abstract

**Background:**

Preeclampsia is 1 of the 3 leading causes of maternal mortality worldwide. Unfortunately, its exact pathogenesis is still unclear. Published metabolomic and gene expression analyses point to coenzyme A (CoA) restriction in the placenta as a factor underpinning the observed complications of preeclampsia, but this hypothesis has never been tested.

**Objective:**

This pilot study aims to discover evidence supporting the CoA-restriction hypothesis through 2 avenues. The first of these involves developing a procedure for the quantitative determination of metabolites to discover if harmful metabolites are elevated in patients with preeclampsia, while the second seeks to emulate the onset of CoA restriction in cultured cells.

**Methods:**

This manuscript provides a rationale and a protocol for a clinical study and laboratory experiments to test the hypothesis. The methods have 3 key aspects. Phase 1 comprises optimization of assays of 5 key metabolites arising from CoA restriction, namely L-leucine, ketoisovalerate, ketodeoxycholate, oleic acid, and sphingosine-1-phosphate. Phase 2 comprises recruitment of patients to obtain serum samples to measure the metabolites, and phase 3 comprises culturing and treating trophoblast cells to induce CoA restriction and test the effects of the metabolites on the cells. Patients with preeclampsia and healthy controls will be recruited based on World Health Organization criteria for preeclampsia. Exclusion criteria include multiple pregnancies, premature rupture of membranes, and various medical complications. Blood samples will be collected and analyzed using high-performance liquid chromatography/mass spectrometry (HPLC/MS) to quantify key metabolites associated with CoA restriction. For trophoblast cell studies, BeWo cells will be cultured under conditions likely to induce CoA restriction, including hypoxia and human chorionic gonadotropin supplementation, and will also be treated with the key metabolites to determine what effect they might have. Cell viability, apoptosis, energy metabolism, and gene expression (focusing on genes involved in CoA synthesis and metabolism) will be assessed. Statistical analysis will involve 2-tailed *t* tests or Mann-Whitney *U* tests to compare metabolite concentrations between patients with preeclampsia and controls. A correlation matrix will be used to explore associations between metabolite levels and patient characteristics.

**Results:**

Institutional review board ethics approval has been obtained for this study. Patient recruitment started April 1, 2025. The 5 metabolites have been purchased in synthetic form and used to optimize the HPLC/MS assays in preparation for receiving blood samples. The trophoblast cell-line culture is being optimized.

**Conclusions:**

The findings of this study will demonstrate that key metabolite concentrations can be quantified using HPLC/MS and indicate if CoA restriction is associated with preeclampsia. If so, this provides a significant, novel avenue for research into the treatment and prevention of the disease.

**International Registered Report Identifier (IRRID):**

PRR1-10.2196/66202

## Introduction

### Background

Preeclampsia is a major pregnancy-related disorder that affects approximately 3% to 10% of pregnancies worldwide [1], and it is also 1 of the 3 leading causes of maternal morbidity and mortality [2]. Women affected by preeclampsia usually present with new-onset hypertension and proteinuria developing during the second half of pregnancy, though presentation can vary. In severe cases, organ damage to the kidney and liver occurs, with hematological involvement and, if left to progress, women will experience neurological complications including seizures or eclamptic fits [3,4]. The babies of women with preeclampsia are at increased risk of intrauterine growth retardation, placental abruption, preterm rupture of membranes, premature delivery, and death [5]. It is therefore important to further investigate the pathophysiology of preeclampsia and potential drug targets, with the aim of reducing maternal mortality and morbidity worldwide.

Unfortunately, the exact pathogenesis of preeclampsia is still unclear, although it is generally thought that dysfunction of the maternal endothelium caused by systemic inflammation and oxidative stress plays a critical role in the development and progression of the disease [6]. A multiomics analysis of 7 untargeted studies has shown that a reduced level of placental coenzyme A (CoA) explains the change in concentration of at least half (26/51) of perturbed plasma metabolites in the first trimester of women who go on to be affected by preeclampsia [3]. Also, gene-expression changes indicate a reduced ability to synthesize CoA from vitamin B5, reduced expression of genes for enzymes consuming free cytosolic CoA, and elevated expression of enzymes releasing CoA from thioesters [3]. CoA is a fundamental carrier of acyl groups in many metabolic pathways that are critical for the cellular reactions involved in energy metabolism and branched-chain amino acid degradation [3]. Therefore, any disruption in the synthesis of this essential cofactor can lead to a disruption in energy metabolism and have significant physiological consequences, including preeclampsia.

### Perturbed Metabolites

**Figure 1:**
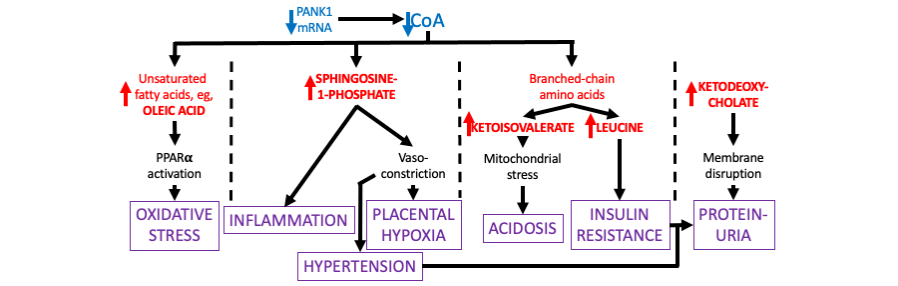
Clinical consequences of placental coenzyme A (CoA) restriction. Text in blue, red, black, and purple respectively denotes reduced concentrations, elevated concentrations, physiological conditions, and pathological states. Red and blue arrows respectively represent increased and decreased molecular concentrations. Black arrows depict the chains of consequence leading to disease states. The dashed lines separate different areas of metabolism. Pantothenate kinase 1 (PANK1) is the initial and regulatory enzyme in CoA synthesis from vitamin B5. This leads to elevated levels of unsaturated fatty acids, sphingosine-1-phosphate, and interruption of branched-chain aliphatic amino acid degradation. PPARα: peroxisome proliferator activator receptor α.

In this context, CoA restriction leads to elevated levels of potentially harmful metabolites, even in the first trimester. These include dietary unsaturated fatty acids, sphingosine-1-phosphate, branched-chain amino acids and their keto acids, and ketodeoxycholates (Figure 1) [7]. Unsaturated fatty acids, including oleic acid, bind to peroxisome proliferator activator receptor α (PPARα), promoting peroxisome formation. Transcriptomic data reveals elevated expression of genes for peroxisomal proteins. Particularly under CoA restriction, this can explain the known oxidative stress observed in preeclampsia. Depending on the receptors to which it binds, sphingosine-1-phosphate exerts various physiological effects, including cell survival, angiogenesis, vasoconstriction (inducing hypertension), macrophage recruitment, inflammation, and programmed cell death [8].

The plasma levels of 6 amino acids are significantly elevated, including the branched chain amino acids valine, isoleucine, and leucine [3]. The latter can cause insulin resistance by blocking signaling via the mammalian target of rapamycin complex 1 (mTORC1), providing an explanation for the sometimes-observed elevated glucose level and possibly contributing to the proteinuria typical of preeclampsia. The equivalent ketoacids of these amino acids, as well as some of their intermediate degradation products, are elevated (indicative of CoA restriction) and are known to cause mitochondrial stress, acidosis, and neurotoxicity, especially 2-oxomethyl butanoic acid (also known as ketoisovaleric acid), 3-hydroxyisovaleric acid, and 3-hydroxybutanoic acid. Ketodeoxycholates are secreted from and recycled by the liver to disrupt ingested membranes. At high enough levels in plasma, they can disrupt the cell membranes of many organs, including the kidney (leading to proteinuria), blood cells, and the liver (contributing to hemolysis, elevated liver enzymes, and low platelets syndrome [HELLP]). The past multiomics study mentioned above also identified adverse positive-regulatory feedback loops [3]. In placental cells, they can cause a progressive increase in the concentration of the harmful metabolites that ultimately causes preeclampsia. Finally, it should also be noted that oleic acid, sphingosine-1-phosphate, and ketoisovaleric acid are also members of a predictive biomarker set for preeclampsia [7].

### Placental Gene Expression Changes in Preeclampsia

A meta-analysis [9] revealed that the expression of over 7600 genes in the placenta is altered in preeclampsia. To test the hypothesis of CoA deficiency, the present study will focus on genes influencing the synthesis pathway of CoA and the above metabolites, as well as on insulin resistance. The pantothenate kinase 1 (PANK1) gene, whose transcription levels are low, and even lower in preeclampsia, encodes the crucial first step in CoA synthesis. Programmed cell death is thought to repress PANK1, which at this early stage is probably due to hypoxia. However, expression of the gene for the final step of CoA synthesis, dephospho-CoA kinase (DCAKD), is elevated. In preeclampsia, lipoprotein lipase (LPL) gene expression is elevated, which increases the release of fatty acids from chylomicrons, including oleic acid. The initial step in the pathway to sphingosine-1-phosphate is catalyzed by serine palmitoyl transferase, whose activity is increased by the elevated expression of its activator (serine palmitoyltransferase small subunit A [SPTSSA]).

Branched-chain amino acids are usually degraded within mitochondria. However, in preeclampsia, the placenta also overexpresses the gene for cytosolic branched-chain amino-transferase 1 (BCAT1), allowing ketoisovalerate to be produced even in the cytosol. In mitochondria, the next step of branched-chain amino-acid degradation is impeded by the elevated expression of an inactivating protein kinase (branched-chain ketoacid dehydrogenase kinase; BCKDK), whose expression should otherwise be repressed by the activated PPARα. Finally, the expression of phosphatidyl choline transfer protein (PCTP) is elevated to enable free fatty acids to enter mitochondria, but this protein also blocks insulin signaling via mTORC1, and it is of interest for this reason.

### Trophoblast Biology

Preeclampsia can occur in the absence of a fetus but in the presence of trophoblast tissue with hydatidiform moles [10], indicating a crucial role for trophoblasts. Indeed, trophoblasts play a principal role in placental development [11]. Their cell lineage begins with cytotrophoblasts, which are the initial stem cells that proliferate in the first and second trimesters and go on to form daughter cells with defined roles. Those in the cell column first specialize into extravillous trophoblasts, from which 2 further cell types arise. Interstitial extravillous trophoblasts anchor the growing placenta to the uterus, while endovascular extravillous trophoblasts invade the maternal spiral arteries, replacing the endothelial lining to allow low-pressure perfusion of maternal blood through a lumen to the maternal veins.

This lumen is filled with chorionic villi, providing a very large surface area permitting exchange of gases and biochemicals between the maternal and fetal vascular systems. These villi have two cell layers. Cytotrophoblasts line the layer next to the fetal blood, while multinucleated syncytiotrophoblasts coat the surface next to the maternal blood. The latter are formed by trophoblast-cell fusion, which is promoted by human chorionic gonadotrophin (hCG) and insulin-like growth factors [12,13].

Trophoblasts have been grown in cell culture for 60 years. Apart from primary cell cultures and immortalized cell lines, there are also several gestational choriocarcinoma cell lines [14]. Of the latter, BeWo cells are derived from a cytotrophoblast and widely used as a model because they can also fuse to form a syncytium [15]. A recent transcriptomic comparison of cell lines indicated that none were very similar to various trophoblasts extracted by cell sorting, though BeWo cells were the closest [14]. However, a study focusing on a subset of preeclampsia markers showed that a BeWo-hypoxia model mimics a key pathogenic mechanism of preeclampsia [16]. Programmed cell death plays a critical role in embryonic implantation and placental development, but hypoxia and/or oxidative stress are thought to cause early aberrant trophoblastic cell death [17] that eventually results in preeclampsia.

### Aims of This Study

Prior work indicates that CoA levels are reduced in the first trimester of pregnancies, triggering the rise of harmful metabolites that later cause preeclampsia. It is extremely difficult to test this hypothesis by direct measurement of CoA concentration, because it is bound to many different metabolites and proteins, and obtaining a biopsy from the diseased part of a placenta would be difficult and highly invasive. However, the metabolites generated as a consequence of CoA restriction are readily detected using mass spectroscopy, even though they are a mixture of hydrophilic and hydrophobic molecules. Hence, this pilot study aims to uncover evidence supporting the CoA restriction hypothesis through 2 avenues. The first of these is to provide the proof of concept of a methodology to quantify the metabolites (specified in Figure 1) and then compare the levels in patients with preeclampsia versus matched controls. The second seeks to emulate the onset of CoA restriction in cultured cells.

**Figure 2:**
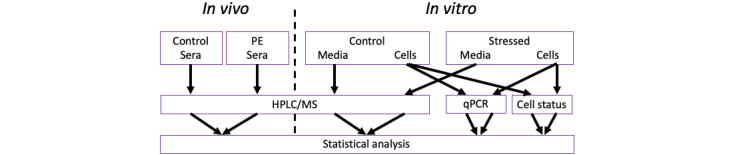
Flow of materials and data through the CoA restriction study. The in vivo and in vitro components are demarcated by the dashed line. The top row of boxes indicates the control and disease or stressed material that is fed into the middle blocks, denoting laboratory techniques. The output data from these assays are then subject to statistical analysis to identify significant changes. Cell status refers to assays of cytotoxicity, apoptosis, and energy metabolism. HPLC/MS: high performance liquid chromatography/mass spectrometry; PE: preeclampsia; qPCR: quantitative polymerase chain reaction.

Hence, this article outlines a 3-phase protocol (protocol version 1.1, May 23, 2024) for clinical samples and cell culture studies to screen for the indirect effects of CoA restriction (Figure 2). Initially, laboratory methods will be developed to quantify levels of the harmful metabolites in serum. Then, blood samples from patients with preeclampsia and matched controls will be taken to determine if the predicted harmful metabolites are elevated in patients with the disease. In parallel, trophoblast cells will be stressed to induce CoA restriction, and then investigated in 3 ways. The first will determine if the harmful metabolites appear in the cell culture medium, while the second will study the effect of these metabolites on otherwise healthy cells and the third will determine the expression level of genes known to be affected by the disease (ie, those described in the Placental Gene Expression Changes in Preeclampsia section) to determine if they have significantly changed.

## Methods

### Objectives

This study has 3 objectives: first, develop a set of targeted high performance liquid chromatography/mass spectrometry (HPLC/MS) assays to accurately quantify the concentration of key harmful metabolites in patient blood samples and cell culture media; second, use this set of targeted assays to measure harmful metabolite concentrations in patients with preeclampsia and compare them with healthy controls to determine if they are elevated in preeclampsia; and third, subject the cultured trophoblasts to hypoxia and the harmful metabolites to determine what conditions initiate CoA restriction.

After completion of objective 1, the flow of materials, laboratory investigation, and data analyses will follow the paths outlined in Figure 2.

### Development of Targeted Metabolomic Assays

Oleic acid (Sigma 05508), sphingosine-1-phosphate (Sigma 860492P), L-leucine (Sigma L8000), 3-methyl-2-oxobutyrate (Sigma 198994), and 12-ketodeoxycholic acid (Sigma SMB00913) have been purchased from Sigma Chemical Co. Pure compounds are solubilized and analyzed by direct infusion into an Orbitrap Exploris 480 mass spectrometer (Thermo Fisher Scientific Inc), the abundant intact precursor mass adducts are identified, and the collision energy is optimized for tandem mass spectrometry profiling. The compounds are then diluted in 0.1% formic acid and injected across a 15-cm C18 column to determine their retention time. The resulting precursor-fragment transitions and retention time boundaries thus constitute the specific assays for each metabolite.

The metabolite extraction procedure and absolute quantification limits are then optimized by spiking the metabolite standards into and then extracting them from the commercially available National Institute of Standards and Technology standard plasma matrix.

### Recruitment

Women with preeclampsia will be identified by the obstetricians collaborating with the study from the antenatal clinics and wards at Latifa Hospital in Dubai. Women with preeclampsia who meet the inclusion criteria outlined below will be approached verbally, asking if they would like to participate in this study. A patient information sheet will be provided, and after they provide written informed consent, they will be recruited into the study.

Baseline clinical and laboratory characteristics will be collected from the patients’ medical notes, including obstetric history, booking weight, height, renal and liver function, comorbidities, and smoking history. A data collection form has been designed for this purpose (Multimedia Appendix 1). The collected data will be anonymized and linked to samples by study ID only. Concurrent control participants will be identified following a review of the antenatal clinic lists to seek women with uncomplicated pregnancies who are undergoing routine antenatal care in the same antenatal clinic at Latifa Hospital in Dubai. Controls will be matched for approximate weight, age (5-year age group) and week of gestation. Control participants who meet the inclusion criteria outlined below will also be approached verbally, asking if they would like to participate in this study.

Patient blood samples will be taken at the time of routine phlebotomy for blood tests required as part of clinical care. As such, no adverse events specifically related to the study procedure are anticipated.

Blood samples will be collected in a heparinized EDTA (ethylenediaminetetraacetic acid) 10-ml container and transported immediately on ice to the laboratory for processing, as described in Blood Metabolite Analysis section below. One member of the research team has the responsibility for sample processing to ensure consistency. Metabolite measurements will be conducted by 2 members of the research team.

Preeclampsia will be defined as per the WHO Recommendations for Prevention and Treatment of Pre-eclampsia and Eclampsia [18]; specifically, the onset of a new episode of hypertension during pregnancy, characterized by persistent hypertension (diastolic blood pressure ≥90 mm Hg), and substantial proteinuria (>0.3 g/24 hours or more or a protein level of +2 or above in the urine dipstick test). The gestational week in which the sample is collected will be recorded for all women.

The exclusion criteria will include multiple pregnancy (eg, twins or higher-order gestations), premature rupture of the membranes; chorioamnionitis; oligohydramnios; fetal growth restriction; polyhydramnios; fetal anomalies; local or systemic diseases; tobacco use; and medical complications, including autoimmune disorders (antiphospholipid syndrome, systemic lupus erythematosus, hemolytic uremic syndrome, acute fatty liver of pregnancy, thrombotic thrombocytopenic purpura, and pheochromocytoma); hematologic disease; neurologic disorders; gestational hypertension; hereditary or acquired antithrombin III deficiency; connective tissue disease; diabetes mellitus; chronic hypertension; inflammation; and chronic renal diseases, as well as regular treatment within the last 3 months with aspirin, antihypertensive drugs, nonsteroidal anti-inflammatory drugs, or antibiotics [19].

### Blood Metabolite Analysis

Briefly, 10 ml of blood will be collected in Becton Dickinson (BD) Vacutainer glass plasma tubes with either sodium heparin (366480, green closure) or sodium citrate (366415, blue closure) as an anticoagulant. Blood will be centrifuged at 2400 relative centrifugation force for 20 minutes at 4 °C to separate the plasma. Both the separated plasma and cell-culture medium will be stored at –80 °C. Before the analysis, precipitation of the plasma protein will be done by adding 200 μl of acetonitrile to 100 μl of a thawed plasma sample followed by vortexing. The solution will be then centrifuged at 18,000 relative centrifugation force at 4 °C for 10 minutes and the supernatant dried and reconstituted using 100 μL of water/methanol solution (95:5) containing 0.1% formic acid prior to 2 μL being injected and analyzed with HPLC/MS.

For HPLC/MS, the gradient is held for 1 minute at 5% mobile phase B, and it then progresses from 5% to 10% B over a period of 1 minute, 10% to 25% B over 2 minutes, 25% to 60% B over 2 minutes, and 60% to 95% B over 6 minutes, after which it is held at 95% B for 2 minutes, followed by washing and column equilibration steps. Mobile phase A uses water acidified with 0.1% formic acid, and mobile phase B uses 80% acetonitrile-water acidified with 0.1% formic acid. The mass spectrometry analyses will be performed using the targeted assay settings described above. Samples will then additionally be analyzed with untargeted methods developed for untargeted metabolomics [20-25].

### Trophoblast Growth and Treatments

First, human placental–derived choriocarcinoma trophoblastic (BeWo) cells will be obtained from the American Type Culture Collection. In the unlikely event that this cell line is ineffective, others are available for investigation. The cells are cultured in Dulbecco’s modified Eagle’s medium/12 Ham’s media (Sigma; catalog number D8437; 500 ml) supplemented with 10% fetal bovine serum (Sigma; catalog number F9665; 500 ml) and 1% penicillin streptomycin (Sigma; catalog number P4333; 100 ml). A total of 1 × 10^6^ cells are dispensed into a culture flask, and the cells are incubated at 37 °C in a 5% CO_2_ incubator for cellular attachment.

The first set of treatments will involve a solvent control and cells subjected to hypoxia, hCG supplementation (obtained from ProspecBio; catalog number HOR-250; 5 mg), or both to stimulate syncytialization. Cytotoxicity assays will be carried out as described below. The second set of treatments will involve addition of the abovementioned harmful metabolites at a range of concentrations and screened for cytotoxicity. Cells showing evidence of stress in microscopy will be tested for cytotoxicity, apoptosis, and energy metabolism, as described below. In such cases, the culture medium will be used for metabolite screening, as outlined above. The cells will be harvested to determine the expression level of 7 key genes, as described below, and stored frozen for transcriptomic analysis in a future project.

### Trophoblast Cell Studies

Cell viability will be determined using a standard MTT (3-(4,5-dimethylthiazol-2-yl)-2,5-diphenyltetrazolium bromide) assay kit (Abcam, catalog number ab197010; 250 tests) [26] according to the manufacturer’s protocol. Energy metabolism will be studied by measuring glucose uptake and the adenosine diphosphate (ADP) to adenosine triphosphate (ATP) ratio, as described previously in the literature [27]. Given the known elevated expression of a membrane scramblase in the placenta of patients with preeclampsia [9], apoptosis and necrosis will be assessed using the Annexin V-FITC kit (Beckman Coulter), according to the manufacturer’s instructions. Analysis will be performed using a BD FACsAria flow cytometer, collecting a total of 100,000 events. For cultures that appear stressed, metabolite concentrations will be determined by extracting metabolites from the culture medium and analyzing them with HPLC/MS in the same way as described above for the plasma samples.

### Gene Expression Profiling

Primers for the BCAT1, BCKDK, DCAKD, LPL, PANK1, SPTSSA, and PCTP genes will be determined using the web tool provided by Thermo Fisher Scientific. After treatment, the BeWo cells will be gently washed with precooled phosphate-buffered saline. Total RNA will be extracted using the RNeasy mini kit (Qiagen). Quantitative polymerase chain reaction (qPCR) will be performed with a real-time PCR detection system and software (Applied Biosystems).

### Data and Statistical Analysis

The patient metabolite-concentration data will be examined to determine differences between the preeclampsia and healthy patient samples and to determine if they follow normal distributions. If they do, paired and unpaired 2-tailed *t* tests will be carried out to determine if the concentrations are significantly different. If they are not, then the Mann-Whitney *U* test will be used. These same tests will be used for metabolite concentrations in the in vitro cell cultures. A correlation matrix will be calculated between the metabolite concentrations and patient characteristics, including weight, age, and week of gestation, to determine if there are any associations. In addition to the planned targeted analysis to test our hypothesis, the plasma metabolomes will also be measured with untargeted HPLC/MS, and the data will be analyzed using multiple-testing correction-adjusted *t* tests and partial least-squares discriminant analysis to identify which metabolites are altered by preeclampsia status. Pathway enrichment will be used to identify dysregulated pathways, which will then be compared with previously published data.

### Power

This prospective, cross-sectional, case-control study will include 40 pregnant women, with 20 women diagnosed with, followed up on, and treated for preeclampsia at Latifa Hospital in Dubai, and 20 women (matched for approximate weight, age [5-year age group], type of pregnancy [single or multiple], and week of gestation) with normal pregnancies. This is about half the numbers in previous metabolomic studies of preeclampsia but should be sufficient to produce the variability required given that matched controls are involved. If not, this pilot study will provide the data to carry out a sample-size calculation for a future larger clinical study. The analysis of data from this study will be primarily focused on descriptive statistics and estimates of precision to inform a planned larger validation study. The proposed sample size in our study is also consistent with the findings from a study on the target sample sizes set for pilot and feasibility randomized controlled trials in the United Kingdom [28].

For the HPLC/MS metabolite measurement, assuming a coefficient of variance of 30%, which corresponds to several multiples of observed technical variation, power calculations indicate this study will be 80% powered to detect changes of 27%. The minimum detectable effect size was calculated using G*Power [29,30].

### Ethical Considerations

The study has been granted approval by the institutional research board (IRB) of the Mohammed Bin Rashid University of Medicine and Health Sciences and the Dubai Scientific Research Ethics Committee (reference DSREC-05/2024_06). The approval includes consent forms, patient information leaflets in English and Arabic, and the data collection sheet. In addition, the consent form includes a statement informing patients of the option to opt out from the use of their samples for future secondary studies. The information leaflet and consent process clearly indicate to patients that they may also opt out of the study at any point in the process. All patient data will be anonymized. Patients will be given a study number, which will be used for any analysis. The code for the study ID linking it to the patient will only be available to 2 members of the research team, who will be collecting the clinical data. The clinical data will be deidentified when shared with the remainder of the study team for analysis. Patients’ privacy and confidentiality is included in the IRB approval process and given high importance within the study team. Additional statistical analysis may be sought from statisticians with expertise in scientific methods. The final dataset will remain property of the university sponsoring the research and the study investigators while they carry out the roles identified in the IRB ethics approval. The study is funded by an internal university grant without commercial sponsors or competing interests. No financial compensation will be offered to patients other than any associated travel expenses.

## Results

We have developed a targeted, standards-based HPLC/MS metabolomic assay calibration method. The targeted assays for L-leucine, 3-methyl-2-oxobutyrate, and 12-ketodeoxycholate (using parallel reaction monitoring) have been successfully developed and optimized. Work is presently ongoing to develop optimized chromatography and metabolite extraction and targeted parallel reaction monitoring HPLC/MS methods for oleic acid and sphingosine-1-phosphate. Evaluation of quantitative metrics, such as limits of detection and limits of quantification, are also ongoing. Data analysis has not yet commenced, but we anticipate that initial results for analysis and publication will be available by June 2026. Multimedia Appendix 2 shows a Gantt chart of the project timeline.

## Discussion

### Comparison With Prior Work

Prior metabolomic studies have sought to identify first-trimester biomarkers to predict preeclampsia occurring later in the pregnancy. These biomarkers were investigated by Hodgman et al [3], who took an approach combining their data with that of published gene expression and genome-wide association studies to uncover regulatory mechanisms that can explain the pathophysiology of preeclampsia. Given our prior observations, we hypothesize that the metabolites chosen for quantitative screening should be highly elevated in patients with preeclampsia, to a level that can explain the symptoms observed in the disease. The cell culture experiments aim to uncover what conditions cause elevated levels of the placenta-derived metabolites, with the gene expression profiling showing key transcriptional changes involved in generating these metabolite changes.

### Limitations

This pilot study depends on being able to quantify metabolite levels using our equipment, which can be considered a limitation. If our objectives are achieved, the next potential limitation is the number of patients with preeclampsia and matched controls. At the very least, we expect to identify trends in the data. If the sample size is too small, then the data from this work will form the basis for a power calculation in a future study. Based on the current literature, the trophoblast culture experiments should succeed in revealing elevated levels of the harmful metabolites investigated in this study. If not, the work at least rules out certain possibilities, providing ideas for future studies.

### Conclusions

This pilot study describes a protocol for an ongoing study to develop a technique for quantifying the concentrations of key metabolites using HPLC/MS and then using it to screen cultured trophoblast cells and blood from healthy patients and patients with preeclampsia. This is a step toward testing the hypothesis that CoA restriction early in pregnancy leads to the elevation of metabolites that induce the symptoms of preeclampsia. Although we currently do not have any results to report, the publication of our protocol should aid research collaboration and early feedback. It could also reduce duplication of effort by other research groups. The output from this study has broader implications for the study of preeclampsia, with potential further applications to other significant antenatal diseases, such as hypertension, proteinuria, fetal growth restriction, HELLP, and gestational diabetes.

## Appendix

Multimedia Appendix 1 Approved clinical data collection form.

Multimedia Appendix 2 Gantt chart project and dissemination plan.

Multimedia Appendix 3 Peer review report from MBRU (Mohammed Bin Rashid University) College of Medicine Internal Grant Award 2023-2025.

## Abbreviations

ADP: adenosine diphosphate
ATP: adenosine triphosphate
BCAT1: branched-chain amino-transferase 1
BCKDK: branched-chain ketoacid dehydrogenase kinase
BD: Becton Dickinson
CoA: coenzyme A
DCAKD: dephospho-CoA kinase
EDTA: ethylenediaminetetraacetic
hCG: human chorionic gonadotrophin
HELLP: hemolysis, elevated liver enzymes, and low platelets syndrome
HPLC/MS: high performance liquid chromatography/mass spectrometry
IRB: institutional research board
LPL: lipoprotein lipase
mTORC1: target of rapamycin complex 1
PANK1: pantothenate kinase 1
PCTP: phosphatidyl choline transfer protein
PPARα: peroxisome proliferator activator receptor α
qPCR: quantitative polymerase chain reaction
SPTSSA: serine palmitoyltransferase small subunit A

## Data Availability

All metabolomics data generated as part of this study will be publicly available at the Metabolomics Workbench [31], a website of the NIH Common Fund’s National Metabolomics Data Repository, where it will be assigned a unique project ID and accessed directly via its project DOI.
