# Supplementary material for: Investigating the Role of Coenzyme A Restriction in the Pathophysiology of Preeclampsia: Protocol for a Combined Patient Screening and Laboratory Study
Source: JMIR Res Protoc. 2025 Oct 3;14:e66202. doi: 10.2196/66202 (PMC12534760; doi:10.2196/66202)
Supplement: Multimedia Appendix 1 [file resprot_v14i1e66202_app1.docx]

**Project Title Investigating the role of Co-enzyme A restriction in the pathophysiology of Pre-eclampsia**

**Data Collection Form 2: Demographic and Clinical Data**

| Patient MBRU Research Number |  | Date  (dd/mm/yyyy) |  |
| --- | --- | --- | --- |
| Patient Hospital Research Number |  | Collected by |  |
| Hospital Name | Latifa Hospital |  |  |

| Date of Birth (dd/mm/yyyy) |  |
| --- | --- |
| Informed consent obtained (Yes/No) |  |
| Date and time of sample collection. |  |
| Parity (number) |  |
| Live births |  |
| Miscarriages |  |
| Induced abortions |  |
| History of pre-eclampsia yes/no |  |
| Weeks of Gestation (ultrasound dating) |  |
| First day of Last Menstrual Period (dd/mm/yyyy) |  |
| Estimated date of delivery (dd/mm/yyyy) |  |
| Pre-eclampsia or Control participant |  |
| If control did woman develop pre-eclempsia after sample collection |  |
| Date of diagnosis of Pre-Eclampsia (dd/mm/yyyy) |  |
| Systolic Blood pressure (BP) at diagnosis |  |
| Diastolic BP at diagnosis. |  |
| Any pregnancy complications so far apart from pre-eclampsia (Yes/No). |  |
| If yes to the question above, please state. |  |
| Other (not pre-eclampsia) past medical history  including pre- pregnancy diagnosis of hypertension | Pre-pregnancy Co-Morbidity   a. Hypertension     b. Diabetes     c. Autoimmune disorders     d. Thyroid disorders     e. Kidney disease     f. Other (please specify) |
| family history of pre-eclampsia |  |
| Prescribed medication at time of sample collection |  |
| Smoker (Yes/No) |  |
| Alcohol (Yes/No) |  |
| Height |  |
| Weight (now) |  |
| Weight at booking |  |
| Week gestation at booking |  |
| Urine Albumin:creatinine ration, Urine Protein:Creatinine ratio or 24 hr urine protein at diagnosis if available. State units |  |
| Liver function tests at diagnosis (including albumin) | ALT, AST, Bilirubin, INR |
| Platelet count at diagnosis |  |
| Medication patient taking at time of sample withdrawal | a. Antihypertensives:      b. Aspirin or anticoagulants:      c. Other (please specify): |
| Smoker | Current/Ex-smoker/Non smoker |
| Pregnancy outcome (to be added later) |  |
| Presenting signs and symptoms |  |
| Complications of pre-eclampsia- data at time of sample collection and at end of pregnancy | Visual disturbance  Stroke  Pulmonary edema  Clotting derangement  Liver derangement  Impaired renal function  IUGR  Other |
| For Research Team at MBRU Use only  Metabolite levels | 1. Shingosine-1-phosphate  2. Oxo-methyl butanoic acid  3. Oleic acid  4. Leucine  5. Ketodeoxycholic acid |
