# Supplementary material for: Investigating the Role of Coenzyme A Restriction in the Pathophysiology of Preeclampsia: Protocol for a Combined Patient Screening and Laboratory Study
Source: JMIR Res Protoc. 2025 Oct 3;14:e66202. doi: 10.2196/66202 (PMC12534760; doi:10.2196/66202)
Supplement: Multimedia Appendix 2 [file resprot_v14i1e66202_app2.pptx]

## Slide 1
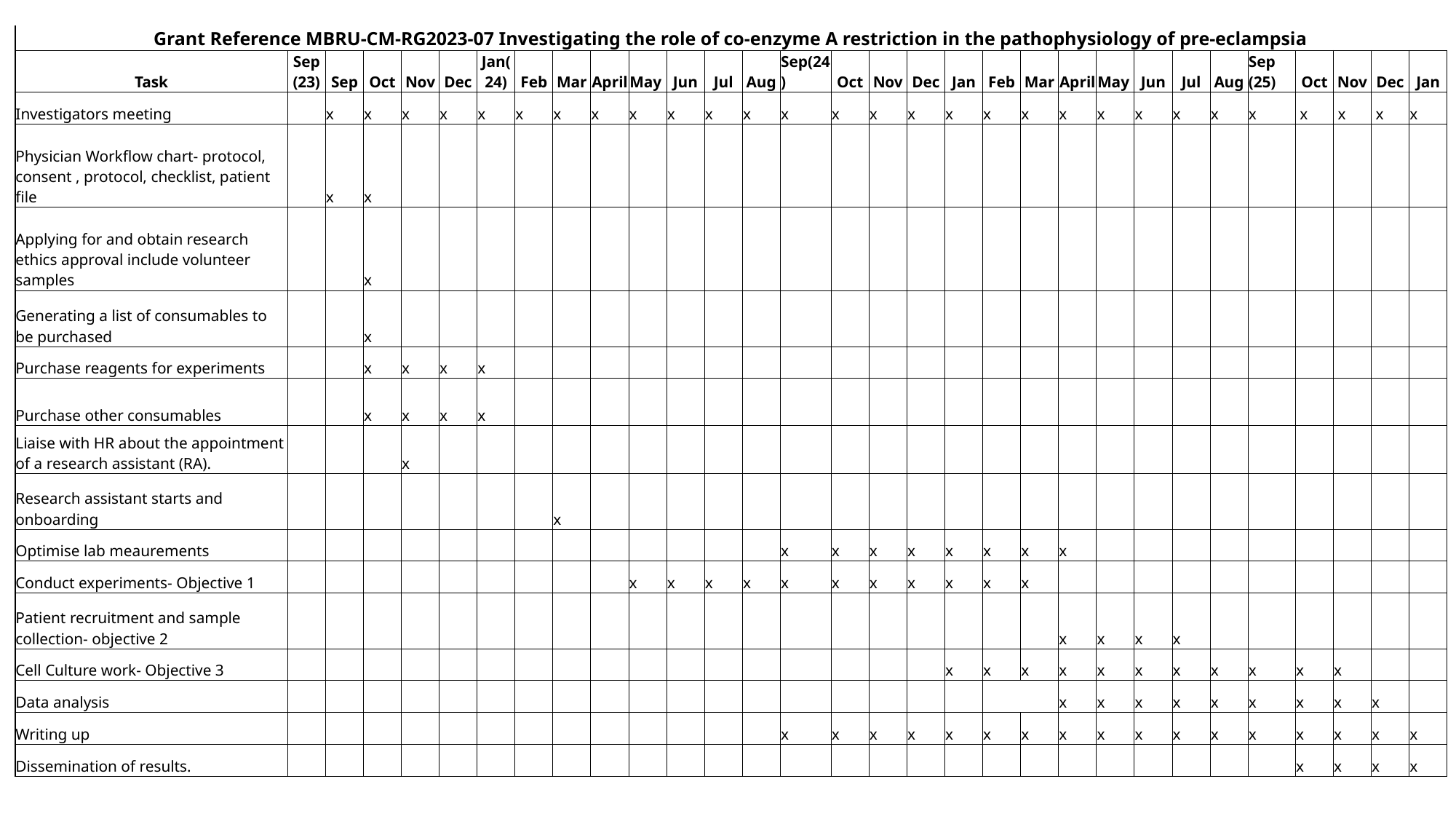

| Grant Reference MBRU-CM-RG2023-07 Investigating the role of co-enzyme A restriction in the pathophysiology of pre-eclampsia | | | | | | | | | | | | | | | | | | | | | | | | | | | | | | |
| --- | --- | --- | --- | --- | --- | --- | --- | --- | --- | --- | --- | --- | --- | --- | --- | --- | --- | --- | --- | --- | --- | --- | --- | --- | --- | --- | --- | --- | --- | --- |
| Task | Sep (23) | Sep | Oct | Nov | Dec | Jan(24) | Feb | Mar | April | May | Jun | Jul | Aug | Sep(24) | Oct | Nov | Dec | Jan | Feb | Mar | April | May | Jun | Jul | Aug | Sep (25) | Oct | Nov | Dec | Jan |
| Investigators meeting | | x | x | x | x | x | x | x | x | x | x | x | x | x | x | x | x | x | x | x | x | x | x | x | x | x | x | x | x | x |
| Physician Workflow chart- protocol, consent , protocol, checklist, patient file | | x | x | | | | | | | | | | | | | | | | | | | | | | | | | | | |
| Applying for and obtain research ethics approval include volunteer samples | | | x | | | | | | | | | | | | | | | | | | | | | | | | | | | |
| Generating a list of consumables to be purchased | | | x | | | | | | | | | | | | | | | | | | | | | | | | | | | |
| Purchase reagents for experiments | | | x | x | x | x | | | | | | | | | | | | | | | | | | | | | | | | |
| Purchase other consumables | | | x | x | x | x | | | | | | | | | | | | | | | | | | | | | | | | |
| Liaise with HR about the appointment of a research assistant (RA). | | | | x | | | | | | | | | | | | | | | | | | | | | | | | | | |
| Research assistant starts and onboarding | | | | | | | | x | | | | | | | | | | | | | | | | | | | | | | |
| Optimise lab meaurements | | | | | | | | | | | | | | x | x | x | x | x | x | x | x | | | | | | | | | |
| Conduct experiments- Objective 1 | | | | | | | | | | x | x | x | x | x | x | x | x | x | x | x | | | | | | | | | | |
| Patient recruitment and sample collection- objective 2 | | | | | | | | | | | | | | | | | | | | | x | x | x | x | | | | | | |
| Cell Culture work- Objective 3 | | | | | | | | | | | | | | | | | | x | x | x | x | x | x | x | x | x | x | x | | |
| Data analysis | | | | | | | | | | | | | | | | | | | | | x | x | x | x | x | x | x | x | x | |
| Writing up | | | | | | | | | | | | | | x | x | x | x | x | x | x | x | x | x | x | x | x | x | x | x | x |
| Dissemination of results. | | | | | | | | | | | | | | | | | | | | | | | | | | | x | x | x | x |
