# Supplementary material for: Investigating the Role of Coenzyme A Restriction in the Pathophysiology of Preeclampsia: Protocol for a Combined Patient Screening and Laboratory Study
Source: JMIR Res Protoc. 2025 Oct 3;14:e66202. doi: 10.2196/66202 (PMC12534760; doi:10.2196/66202)
Supplement: Multimedia Appendix 3 [file resprot_v14i1e66202_app3.pdf]

Dr. Reem Aljayousi

College of Medicine, MBRU

MBRU College of Medicine Internal Grant Award 2023-2025

Grant Reference No: **MBRU-CM-RG2023-07**

Title: **Investigating the role of co-enzyme A restriction in the pathophysiology of pre-eclampsia**

Feedback and Comments

First Reviewer

Objective 1

Define the study population. Will the blood be obtained from healthy volunteer (e.g. non-pregnant females or non pre-eclampsia pregnant females? Why not use the same cohort as for Objective 2?

How many samples will be analysed?

Does the 5 metabolites to be measured indicate CoA restriction or are they rather relate to/predictive of Preeclampsia?

What about measuring metabolites in the CoA synthesis pathway from Pantothenate up to Dephospho-CoA. Accumulation could indicate non-conversion to CoA.

Furthermore, a reduction e.g. Dephosphocoenzyme A Kinase levels could also indicate to a lower restriction in synthesis.

Why not also investigate the Citric Acid Cycle by measuring Pyruvate, Acetyl-CoA and Citrate for example?

What if Objective 1 is inconclusive – will you not proceed since Objective 2 is dependent?

Objective 2

I see no reason for separating Objective 1 and Objective 2.

A sample size calculation would have been preferred, but the justification is acceptable.

Objective 3

No indication in terms of the quantum of experiments/cultures/repeats.

General

Define: HELLP - Hemolysis, Elevated Liver enzymes and Low Platelets

Correction: Ethics to be obtained from MBRU-IRB and DHA

Comments:

As stated, this is a proof-of-concept project and therefore rather ambitious, with a risk of "failure". However, there is enough justification from the literature to warrant this approach and line of investigation.

The project includes both human and tissue culture and is relating clinical medicine to wetlab research, which has the potential for translational research.

The PI does not have a significant track record of original/basic medical science research. However, the research team is rather diverse and brings a lot of expertise. What I sense is a lack of experience in Tissue Culture.

Budget – I am not sure if the consumable amount would be sufficient for LC-MS as well as TC, while it will be difficult to employ an experienced RA for 2-years on the amount set aside for personnel.

دبي الصحية
DUBAI HEALTH
جامعة محمد بن راشد للطب والعلوم الصحية
Mohammed Bin Rashid University of Medicine and Health Sciences

Final recommendation:

I am of the opinion that this novel project should be supported.

Second Reviewer

This research proposal highlights a major global cause of maternal morbidity and mortality. The investigators of this study hypothesize that a metabolic dysregulation associated with co-enzyme A is responsible for the underlying pathogenesis of pre-eclampsia. The study outlines a translational prospective approach.

Please see below, specific comments that need to be addressed:
Comment 1: As part of objective 1 of this study, it is mentioned that "Since quantification of CoA is practically difficult, we will determine whether metabolites indicating CoA restriction can be measured in human serum and what differences (levels) are present". According to the investigators, Objective 2 (i.e., measure levels of serum markers of CoA restriction in 20 women in the 2nd or 3rd trimester of pregnancy) is dependent on the achievement of Objective 1. In the case that metabolites cannot be measured in human serum, how will this be addressed? Is there an alternative to Objective 2 since it is dependent on Objective 1?

Comment 2: It has been widely accepted that dysfunction of the maternal endothelium plays a role in development of pre-eclampsia. This research will not focus on maternal endothelium, however how will its possible link to this study be accounted for/eliminated in the results?

Comment 3: Latifa Women and Children Hospital (LWCH) is a DHA hospital, hence ethical approval received from MBRU-IRB should also cover this facility. Investigators are encouraged to clarify this process as this will affect the study timeline.

Comment 4: It is understood that patient recruitment and serum collection will be done at LWCH. However, it is not clear at which site the experimental part of the study will be conducted as no expenses have been listed under the equipment category.

Comment 5: While investigators state that the power calculation was not performed due to the study being that of a proof-of-concept, it is recommended that they clarify the proposed sample number (n = 40) with a biostatistician, particularly because the study is prospective in nature, with no realistic timeline provided.

Comment 6: The Research Plan section is not reader-friendly and proves difficult to follow. It is recommended that investigators reorder this section as follows: Study Design and Patient Recruitment; Experiments (e.g., Cell Culture Measurement of cytotoxicity etc. – these should be mentioned sequentially and linked to study objectives) Statistical Analysis; Expected Results; and Potential hurdles/Limitations.

Comment 7: A flow diagram, depicting the overall blueprint of the project, should be included in the Research Plan section as this will improve readability.

دبي الصحية
DUBAI HEALTH
جامعة محمد بن راشد للطب والعلوم الصحية
Mohammed Bin Rashid University of Medicine and Health Sciences

Comment 8: In light of comments 6 and 7, sample number and specific experiments should be stated in the “Methods” section of the abstract. At present, it most conveys the study objectives.

دبي الصحية
DUBAI HEALTH
جامعة محمد بن راشد للطب والعلوم الصحية
Mohammed Bin Rashid University of Medicine and Health Sciences
